# Principles, applications, and future of artificial intelligence in dermatology

**DOI:** 10.3389/fmed.2023.1278232

**Published:** 2023-10-12

**Authors:** Jesutofunmi A. Omiye, Haiwen Gui, Roxana Daneshjou, Zhuo Ran Cai, Vijaytha Muralidharan

**Affiliations:** ^1^Department of Dermatology, Stanford University, Stanford, CA, United States; ^2^Department of Biomedical Data Science, Stanford University, Stanford, CA, United States

**Keywords:** dermatology, artificial intelligence (AI), large language models (LLM), machine learning, melanoma, federated learning

## Abstract

This paper provides an overview of artificial-intelligence (AI), as applied to dermatology. We focus our discussion on methodology, AI applications for various skin diseases, limitations, and future opportunities. We review how the current image-based models are being implemented in dermatology across disease subsets, and highlight the challenges facing widespread adoption. Additionally, we discuss how the future of AI in dermatology might evolve and the emerging paradigm of large language, and multi-modal models to emphasize the importance of developing responsible, fair, and equitable models in dermatology.

## 1. Introduction

Recent advancements in artificial intelligence (AI) have fueled an interest in the utility of AI models in medicine (1). These models range from computer vision models that can interpret medical images (2) to large language models (LLM) that have capabilities for analyzing text data (3, 4) to multi-modal models that take both images and text as input (5). These AI models now have the capacity to analyze unstructured data such as clinical notes (3, 6), identify novel correlations in large datasets (7), and generate synthetic image data for improving model training (8, 9).

One medical specialty poised to benefit from these emerging AI technologies is dermatology. Its inherent visual diagnostic process, combined with an increasing volume of clinical photographs, dermoscopy images, and electronic health records (EHR) data (10) underscores its suitability for AI-augmented patient care. Moreover, the shortage of specialists-3.65 dermatologists per 100,000 people in the US (11, 12) and limited access to dermatological services in many regions (13, 14) provides a compelling case for augmented intelligent systems to help bridge this access gap (15). However, clinical integration of AI in dermatology workflow remains challenging. As novel medical applications arise, they also unveil problems that necessitate further research.

In this paper, we present a comprehensive overview of the fundamental principles of AI methodology as applied to dermatology, diving into categories and training approaches. Special emphasis is placed on the role of AI in the diagnosis and prognostication of an array of skin conditions. We also address the limitations of the AI models used in dermatology, notably issues of generalizability, bias, and explainability. Finally, we examine what the future might hold for dermatology-AI, while highlighting some research opportunities to help improve real-world utility of AI models. Our goal is to provide the readers with a panoramic view of AI’s principles and evolving role in dermatology, while equipping them with the knowledge to navigate this dynamic field.

## 2. Principles of artificial intelligence

**Figure 1:**
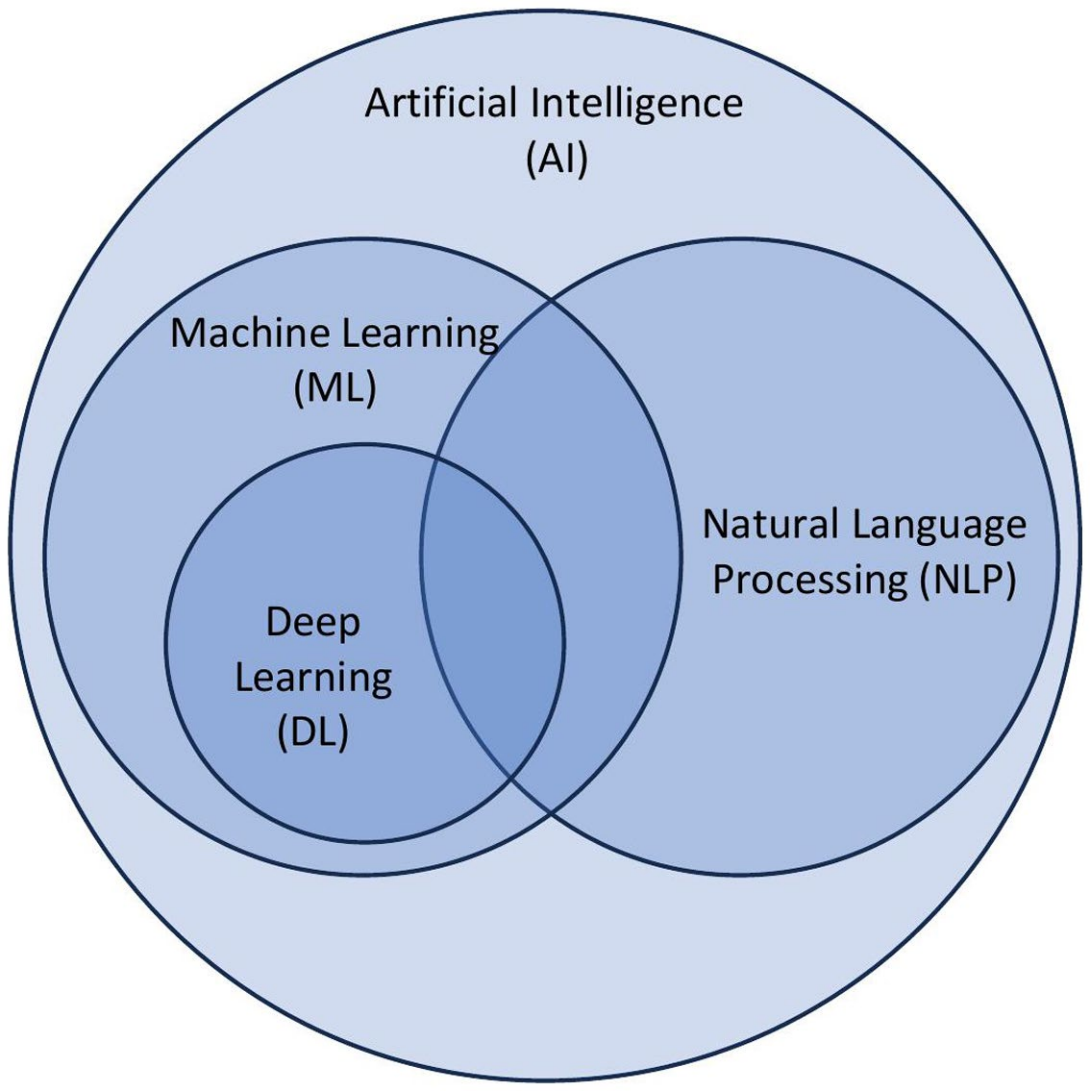
Overview of principles of artificial intelligence. Artificial intelligence (AI) is a broad categorization of algorithms that encompass subcategories including machine learning (ML), natural language processing (NLP), and deep learning (DL).

AI is the ability of a computer system to mimic human cognitive functions and encompasses many computational subfields, including machine learning and natural language processing (Figure 1). Currently, major developments in AI are within the field of machine learning (ML), which are algorithms that make predictions about data without explicit programming. In other words, the machines are “learning” from the data and providing analyses without being explicitly told what features to prioritize. Examples from dermatology include identifying melanomas from clinical images (16), predicting efficacy of biologic therapies in psoriasis (17), and analyzing physician notes in electronic health records to determine focus of atopic dermatitis clinic visits (18).

**Figure 2:**
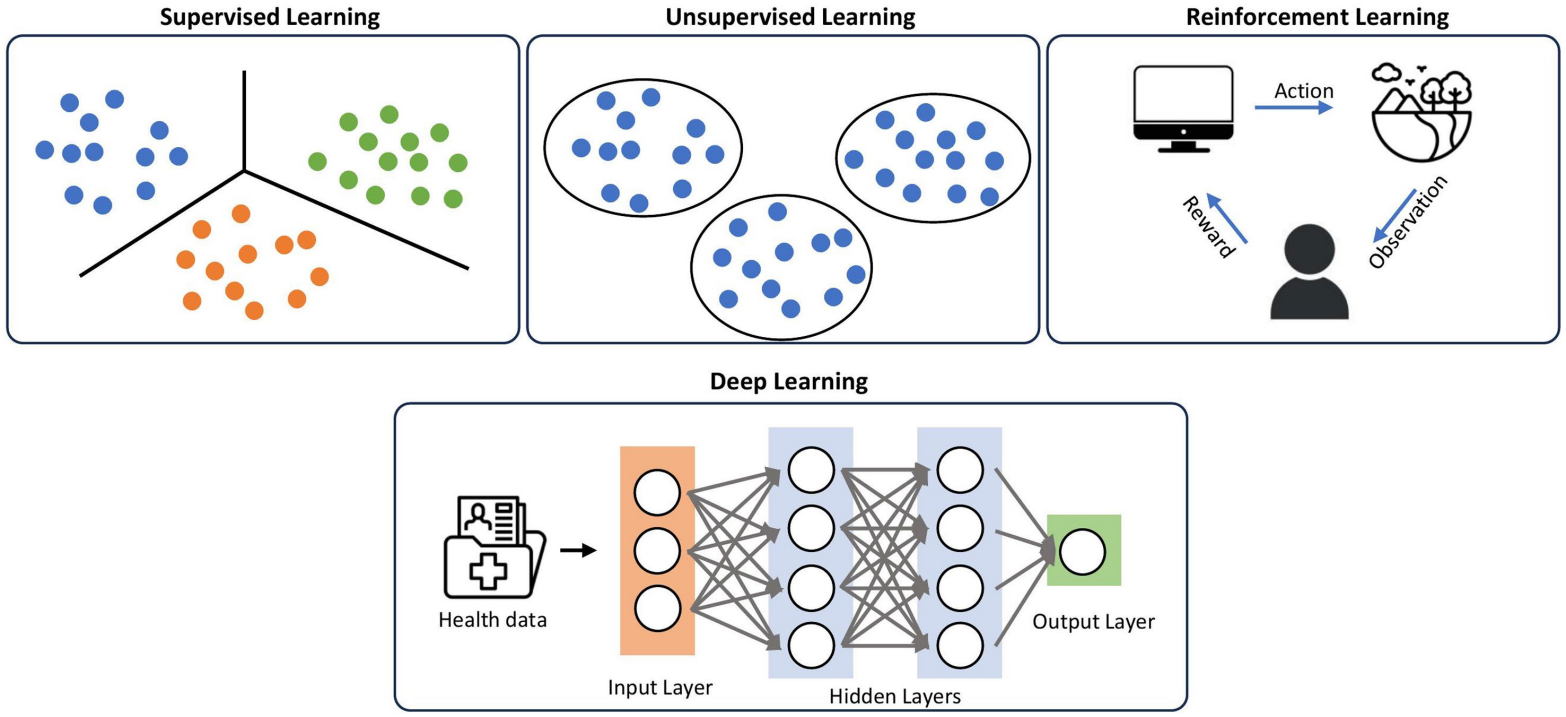
Classifications of machine learning. Supervised learning uses labeled datasets to categorize the data, while unsupervised learning does not have labeled datasets, using patterns and relationships in the data to create categories. Reinforcement learning uses iterative feedback loops to teach the algorithm. DL utilizes representation layers in a neural network to increase abstraction of the data, and employs techniques from supervised, unsupervised, and reinforcement learning.

Deep learning (DL) (19) is a subset of ML that uses algorithms modeled off human neurons that can model complex patterns and relationships in the data. ML techniques prior to the introduction of DL required domain expertise and human engineering to convert raw data into features that the algorithm can understand and detect patterns from. On the other hand, in DL, raw data can be inputted into the algorithm, and the machine is able to create its own representation needed for pattern recognition. These representations are typically arranged in sequential layers, where each layer is inputted into the next layer, increasing the abstraction of the data, collectively known as neural networks (6) (Figure 2). Within DL, there are multiple algorithms that are implemented, including convolutional neural networks (CNN) (20), traditionally used in image processing, and transformer models (21), which are neural networks that learn context and track relationships in sequential data.

Within ML, there are different ways that algorithms can learn, including supervised learning, unsupervised learning, and reinforcement learning (Figure 2). Supervised learning, the most common form of machine learning, uses a labeled dataset to predict results. The algorithm learns to map the input data to the correct output, allowing it to make predictions on unseen data. The algorithm is given the data and the correct answers (ground truths) in a training set, which the algorithm uses to set its weights. Once the algorithm has learned from the training data, its performance is measured against a held-out test set that it has never encountered previously. This category of machine learning includes what most people are familiar with, such as logistic regression, linear regression, etc. Most of the image-based deep learning models in dermatology use supervised learning. Unsupervised learning is training a model on unlabeled datasets, meaning the data input does not have the ground truth. This algorithm aims to find patterns and relationships within the data, such as clustering similar data points together. Finally, reinforcement learning is when the agent (the algorithm) interacts with an environment to achieve specific goals. The agent receives feedback from the user (the human) in the form of rewards or penalties based on its actions, and it learns to optimize its behavior to maximize rewards. Compared to supervised and unsupervised learning, reinforcement learning has no predefined data input, but rather learns from the iterative feedback loops.

Natural language processing (NLP) is a branch of artificial intelligence that focuses on interpreting, analyzing, and generating human language. It combines linguistics with statistics, machine learning, and DL to process human language (22). NLP is generally divided into two subfields-natural language understanding (NLU), and natural language generation (NLG). NLU is focused on determining the understanding of the text, while NLG is focused on generating new text. Recent advancements in large language models, including OpenAI’s (San Francisco, United States) publicly-available ChatGenerative Pre-trained Transformer (ChatGPT) (23), fall under the subfield of NLG.

There are also recent emerging concepts of multimodal approaches, where algorithms are utilizing multiple data types to train their algorithms. Medicine is inherently a multimodal discipline, with clinicians interpreting lab values, clinical notes, radiology images, genomic data, etc. New development has been focused on utilizing the rich diversity of data to build more robust models and algorithms, including Med-PaLM Multimodal (Med-PaLM M) (24), LLaVa-Med (25), Med-Flamingo (5), and MiniGPT-4 (26). These new technologies are built on foundation models (FMs), which are models that are trained on a broad range of unlabeled data that are then adapted (fine-tuned) to specific downstream applications (27). These models can learn from the large amounts of data, and then transfer their learnings to a more specific application, like medicine.

## 3. Applications of AI in dermatology

There has been an abundance of work done to explore artificial intelligence use in all aspects of dermatology (28–30), ranging from skin malignancies to inflammatory skin conditions, to dermatopathology, to text-based analyses. The visual nature of Dermatology lends itself to many advancements that are image-based, though researchers are exploring other multimodal approaches that use patient characteristics and clinical texts. Here, we will provide a broad overview of the different applications of AI in dermatology.

### 3.1. Skin malignancies

Applications of AI in dermatologic malignancies, which have been well described in the literature (31, 32), include identifying and distinguishing between benign nevi and melanoma. Researchers break down images of skin lesions to the pixel level for individual analysis and then utilize the techniques described above to predict and classify malignancies. There have been multiple landmark papers for AI applications in skin malignancies (16, 31, 33), resulting in high sensitivities and specificities when distinguishing malignant from benign lesions. Esteva et al. trained a CNN using a large dataset of over 100,000 biopsy-proven clinical images to determine keratinocyte carcinomas versus benign seborrheic keratoses, and malignant melanomas versus benign nevi (16). Han et al. fine-tuned a previously-built CNN model with clinical images to classify multiple malignancies, including basal cell carcinoma, squamous cell carcinoma, melanoma, etc. (34). There is also an annual international skin imaging competition, which provides publicly accessible dermatology images for researchers to build melanoma-classifying models (32, 35). Aside from identifying the primary lesion, there are also studies exploring metastases. Jansen et al. utilized histological tissue sections of sentinel lymph nodes in their convolutional neural network models to identify presence of metastases with high sensitivity and specificity (36).

### 3.2. Inflammatory skin diseases-psoriasis, dermatitis, and others

Aside from classification of melanomas and other malignant skin conditions, researchers are also exploring the identification and management of inflammatory skin conditions, including psoriasis, dermatitis and acne. Similar to malignancy classification, a majority of work is focused on psoriasis identification and classification through images of skin (37–40), nails (41), and scalp (42) using CNN’s and other DL techniques. In addition to diagnosing psoriasis via image recognition, researchers have utilized machine learning techniques to identify patients with increased risk of associated psoriatic conditions, including psoriatic arthritis (43). Work has also been done to determine the efficacy of psoriasis management by predicting outcomes of biologic therapies by using parameters such as patient demographics, clinical history of psoriasis, treatment history, and presence of other comorbidities (17, 44). These preliminary models could be used to eventually optimize therapy and management for patients. Finally, aside from determining outcomes of current biological treatments, AI techniques have been applied to genomic studies to help with drug target identification and drug repurposing (45), as well as screening for psoriasis biomarkers (46) and gene expression profiling (47).

Similar to diagnostic tasks with psoriasis, many researchers have explored using machine learning algorithms in dermatitis (48), ranging from image-based algorithms (49) to electronic health record text-based algorithms (50). Aside from determining diagnoses, researchers have developed proof-of-concept algorithms using self-reported eczema flare scores, patient demographics and treatment history to predict atopic dermatitis severity, resulting in a biologically interpretable model that focuses on patient’s responsiveness to treatment (51). AI models have also been used to help prevent contact dermatitis by predicting skin sensitization potential and potency of substances (52).

In addition to psoriasis and dermatitis, researchers have developed acne lesion segmentation and evaluation tools (53–55) that can grade acne severity from easily-accessible smartphone images (56). There is also exploration in identifying lichen planus (41), and assessing the severity of hidradenitis suppurativa (57).

### 3.3. Ulcer assessment

One of the primary methods in identifying and classifying skin lesions is segmenting the lesion from the backdrop of normal skin. Multiple studies have explored determining and measuring ill-defined wound boundaries using techniques that simplify images down to the pixel level (58–61). Recent work has been done to apply these techniques into broader hospital systems to predict pressure ulcers (62), with the ultimate goal of pressure ulcer prevention (63, 64). Groups have even explored using body heat maps from pressure mats to identify poor in-bed position posture that could cause pressure ulcers (65). These proof-of-concept works, after validation in clinical trials, may ultimately translate into clinical-assist tools to aid clinicians in the management of ulcers.

### 3.4. Dermatopathology

Beyond identifying diagnoses via clinical images and electronic health record notes, machine learning techniques are being applied in dermatopathology (66, 67). Groups have developed models to classify basal cell carcinoma in digitized Mohs micrographic surgery histology slides to reduce the workload of manually examining these slides (68). Likewise, Hekler et al. used CNNs to aid in histopathologic melanoma diagnoses (69). There have also been studies done to interpret indirect immunofluorescence microscopies to classify bullous dermatoses (70).

### 3.5. Miscellaneous multiclass classification and text-based analysis

To better replicate real-world clinical scenarios of multiple differential diagnoses from a single skin lesion, technologies take a broader approach to solve multi-class classification problems. Many of the problems discussed above were binary classification, where algorithms strived to identify if a lesion was a specific disease or not; multi-class classification presents a more challenging problem with multiple possible diagnoses. Liu et al. created a DL system that provided a differential diagnosis for skin lesions, creating a ranked list of the most likely diagnoses for the skin lesion (71). Taking another multi-class approach, Sitaru et al. have worked to classify body parts from dermatology clinical images, creating body distribution maps for different diagnoses (72).

While dermatology is a visual specialty that focuses on using visual cues for diagnoses, there are aspects of written data that can be used to aid in better understanding questions posed by the research community. Frequently, this written data is unstructured and freeform, using natural human language; to understand and interpret this data, one needs to implement NLP techniques. Researchers have used NLP methods to examine dermatology discussion forums on social media to understand patient perceptions of the field (73). Others conducted analyses of clinical notes in the electronic health records to identify specific topics that providers and patients discuss during clinical visits (18). This analysis provided insights into the lack of documentation of the disease’s impact on a patient’s life, which may ultimately affect management and treatment options.

In addition to understanding natural language, there are also recent advances in technologies that generate new text, including ChatGPT. This technology could be utilized to guide patients, aid clinicians with administrative tasks, educate trainees, etc. Groups are exploring ChatGPT’s ability to generate responses to patient inquiries about melanoma (74), create patient education guides for acne (75), and even triage surgical management of cutaneous neoplasms (76).

### 3.6. Human-AI hybrid models

Given all the innovation that is occurring at the intersection of AI and dermatology, the logical next step is to evaluate the performance of these AI algorithms against clinicians (77, 78). Esteva et al.’s landmark study was the first to compare a DL algorithm against dermatologists, showing that their model was able to match the performance of 21 dermatologists in melanoma classification (16). Others have even shown that, in a group of 58 international dermatologists, many were outperformed by a CNN model (79). Because of the incredible ability of the technology to perform diagnostic tasks, many researchers are exploring ways to incorporate AI in a clinical workflow to help clinicians. There have now been multiple studies creating AI-based assistive tools to aid clinicians in interpreting clinical images. Groups have designed pipelines with the ultimate goal of real-time AI analysis of skin lesions in the clinics (77). Marchetti et al. prospectively assessed the diagnostic accuracy and utility of a melanoma AI algorithm used in real-world clinical settings to help determine the necessity of biopsying a suspicious lesion (33). Han et al. conducted a randomized trial and showed that AI can augment the accuracy of non-expert physicians in the real-world setting (80). With these smaller pilot studies showing promising results, the AI research community may be looking to increase prospective studies and randomized trials to help further assess AI’s application in the real-world clinical setting.

## 4. Limitations and ethical considerations of AI in dermatology

AI research in dermatology is still in its infancy and encounters a myriad of challenges. From biases and lack of interpretability to regulatory hurdles and difficulty in integrating with existing clinical workflows, these issues are complex and need to be tackled before AI can become ubiquitous in clinical practice. Robust, transparent, and equitable AI algorithms are needed in order to truly enhance patient care without introducing new problems.

### 4.1. Datasets

AI algorithms learn by identifying features and patterns found in their training datasets and then use this knowledge to make future predictions. However, the presence of confounders in these datasets can influence the validity of AI algorithms. Confounders are features that may be correlated with the AI algorithm’s outcome through spurious associations. An illustrative example involves the presence of surgical pen markers or rulers on clinical dermatology images. As demonstrated in research by Winkler et al., lesions marked with surgical pen markers are more likely to be classified as malignant by the models (81). This finding is due to the fact that these markings are frequently used during biopsy procedures, which are typically performed on lesions suspected of malignancy. Therefore, an algorithm may incorrectly learn an association between these markers and malignancy, when in fact the markings only indicate which lesions were biopsied, not necessarily those that are malignant. This example highlights the importance of identifying and managing confounders during the training phase of AI models to ensure their accuracy and validity.

Bias in training datasets can also inadvertently perpetuate pre-existing inequities in healthcare. In dermatology, this issue is particularly highlighted by early AI models trained on datasets that predominantly featured lighter Fitzpatrick skin types (I-IV). Daneshjou et al. has shown that some of these existing algorithms tend to underperform when assessed with images of darker Fitzpatrick skin types (V-VI) (15). Fortunately, fine-tuning these original algorithms with a dataset featuring darker Fitzpatrick skin types improved their performance, effectively closing the gap in performance between different skin types. Diverse and equitable data representation in the training dataset is primordial to ensure accurate and fair outputs in AI algorithms.

### 4.2. Image quality and image capturing modalities

Standardizing images in Dermatology AI research is important to preserve data quality. Images can originate from diverse sources, including various devices (e.g., iPhones, Android smartphones, or professional cameras) and with or without the help of diagnostic tools such as dermatoscopes (82). Additionally, the images may be captured under various settings (e.g., at home or in a clinic) and by different individuals (e.g., patients or healthcare providers). These factors result in a highly heterogeneous dataset comprising images of differing quality. Just as human interpretation can be affected by image quality, AI algorithms are equally sensitive. Blurry images with poor lightning have been shown to negatively impact the performance of AI models (83). Simple image manipulations such as rotation can change the output of an algorithm (84). These considerations underscore the importance of establishing robust image capturing standards and Digital Imaging and Communications in Medicine (DICOM) standards similar to those in other medical fields such as cardiology and radiology (85).

### 4.3. Black box

The mechanisms behind traditional medical devices are often transparent and logical in nature. In contrast, AI algorithms appear more mysterious and impenetrable, like a “black box.” This phenomenon makes it difficult for humans to understand its reasoning process and to trust its outputs. Various techniques have been developed by researchers to tackle this problem including saliency maps (e.g., highlighting relevant areas on a picture) and content-based image retrieval (e.g., retrieving similar images from a database based on the query image). As AI penetrates high stake fields such as medicine, it becomes increasingly important to bring transparency and interpretability to AI models.

### 4.4. Implementation

Implementing AI into clinical practice presents a number of challenges that extend beyond technological complexity. The rapid advancement of AI technologies has created a complex landscape of medical-legal challenges regarding its use in the healthcare sector, spanning from concerns about patient consent and data privacy to liability in the event of AI-induced medical errors (86, 87). Scholars and professionals must work collaboratively to devise sound and comprehensive guidance to navigate the ethical and legal intricacies of integrating AI into our healthcare systems (87). Medical AI devices, by their very nature, will evolve as they learn from newly acquired data, a process that may continue long after receiving approval from regulatory bodies such as the Food and Drug Administration (FDA). This continual learning and adaptation, while a strength in many respects, also presents a challenge in ensuring the devices’ sustained reliability and performance over time. Without vigilant monitoring and a robust framework for ongoing validation, there may be unforeseen shifts in the accuracy or effectiveness of these tools, which could potentially negatively impact patient care. Moreover, there is a lack of high quality prospective randomized controlled trials of AI algorithms. While AI holds immense promise in dermatology, the absence of prospective trials hinders the validation of AI models in real-world clinical situations where there will be a diverse photo quality, image capturing modalities and demographically diverse population (33). For these reasons, an AI model validated in a hospital in Asia might not perform similarly in another hospital in North America. Wu et al. have shown that 126 out of 130 FDA approved medical AI devices were trained on retrospective data at the time of their approval (88). Most of the datasets used are not publicly available, thus preventing regulatory bodies and researchers from auditing their algorithms. Future AI models should undergo multi-site validation on a diverse and representative population in order to assess the generalizability of AI models. Furthermore, establishing trust among AI and various stakeholders will be vital in realizing AI’s full potential in the field. While model accuracy is very important, research has shown that dermatologists and patients value the potential of augmented intelligence in dermatology and also put a high priority on the human physician-patient relationship (89, 90).

## 5. Future directions and opportunities

### 5.1. LLMs and the advent of generalist medical AI

In recent months, advanced language models, in the form of chatbots, have gained popularity in medicine (4, 91–93). For dermatology, an extension of these models—Vision-Language Models (VLMs) and multi-modal models—offer immense potential. VLMs are large-scale models adept at associating visual inputs, such as images and videos, with text data (5). Their capabilities span generative tasks (creating new content), retrieval of information, and navigation. Recent studies underscore their impact on dermatology. For instance, Skin-GPT4, a VLM, can provide descriptions and diagnosis from clinical skin lesion photos (94). Further, research by Moor et al. and Tu et al. show the accuracy of VLMs in medical visual question-answering tasks (5, 24). In a related vein, Kim et al.’s study on FMs underscore the capacity of this new class of models to generate accurate skin images annotations (95).

The rapid advancements in this domain have the potential to usher a future of a generalist medical AI (96). These generalist models could be capable of giving approximate diagnoses from clinical photos, generate treatment options, and offer deeper insights into patient data by integrating demographics, visual inspection, and genetic data when applicable. Their potential applications can range from patient chatbots to triage tools (96). Additionally, the inclusion of genetic data could improve the diagnosis of orphan skin conditions. As dermatological datasets expand and computing power increases, FMs are on track to become more accurate and prove utility in dermatology. They could augment the practice of dermatology to provide more precise and holistic care.

### 5.2. Federated learning and the possibility of local models

Medical data, including skin images, are difficult to access largely due to privacy, legal, and the ethical risks associated with sharing health data. Currently, many dermatological images reside in data silos within healthcare institutions all over the world. Also, medical data is hard to collate, and often requires years of planning with significant costs (97). This is even more pronounced in resource-limited settings, where there is less infrastructure to support collection and sharing of data. Since the DL model’s performance significantly improves with more diverse data (98, 99), new approaches are needed to expand model access to more distributed high-quality datasets.

Federated learning (FL) is a concept that enables DL models to be trained on different datasets without the need to leave their original locations (100). In FL, multiple collaborators can train a model on separate institutional datasets. It is an approach that can enable the preservation of data privacy, and it has already demonstrated similar performance—compared to centralizing the data—in fields like radiology and oncology (100). Although in some cases, there have been drawbacks in which the model sometimes memorizes the data inputs (101). Appropriately implemented, FL has the potential to enable fairer and more generalizable dermatology models by incorporating diverse demographics, thereby capturing the nuances in skin conditions across different societies. This is crucial in dermatology where the popular models significantly perform worse on underrepresented skin types of Fitzpatrick IV–VI (15).

Beyond FL, the concept of FMs introduces the possibility of local models. FMs have the distinct capability to learn from unlabeled data and can be adapted for a variety of downstream tasks without the necessity of specific training (96). This characteristic allows FMs to be fine-tuned with local data, from which they can glean insights and achieve impressive performance across diverse tasks. Given that the fine-tuning procedure is more cost-efficient than full-scale training (27), it amplifies the appeal of FMs within institutional contexts. Consequently, dermatology institutions can harness bespoke models attuned to their unique demographics and guidelines. While promising, progress towards this will require resolving data quality, aggregation, and infrastructural challenges. However, these new techniques could be instrumental in building invaluable dermatology-AI models.

### 5.3. Improvements in model architecture and metrics evaluation

Recent years have witnessed notable advancements in the architectures of AI models, leading to enhanced performance across numerous medical tasks as previously discussed. As the industry attracts more investment and data generation surges, new architectures will likely further improve on existing tasks and expand into new areas. However, with these advancements arises a vital question: how should we holistically evaluate these models? While metrics like accuracy, area under the curve are common, comprehensive model evaluation will need to go beyond mere percentages. Clinical value needs to be demonstrated. As reported by Wornow et al., standard evaluations are lacking for evaluating emerging models (102). In addition, many models fail to be evaluated on fairness and transparency metrics, and in many cases there’s no standard for this evaluation frameworks (103). Holistic model evaluation is likely to emerge in the near future as the desire for clinical integration increases. This could include uncertainty, model interpretability, and subpopulation analysis—which is important for dermatology.

Developing these types of model benchmarks will require collaboration among dermatologists, researchers, and patients. We posit that soon, more robust consensus guidelines are likely to emerge.

### 5.4. Regulation, clinical utility, and usability in resource-poor settings

The current rapid model evolution underscores the pressing need for robust regulation (104). Such regulatory measures serve a two-fold purpose: Firstly, they shield the dermatology community from prematurely adopting under-tested models by establishing stringent benchmarks. Secondly, they foster trust, ensuring that AI tools resonate with the foundational clinical values practitioners hold dear. For AI models to achieve widespread adoption, especially in dermatology, they must be both reproducible and generalizable (105). As these models seek to bridge the dermatology access gap, especially in resource-limited settings, generalizability becomes even more pivotal. Also, standardizing the data collection process is another important factor towards optimizing model training and thus, performance. As highlighted in the position statement by the American Academy of Dermatology Augmented Intelligence working group, research in the dermatology-AI space needs to be directed towards prospective and randomized clinical trials that rigorously vet models before deployment (29, 106). Also, although most DL models are in the form of “black-boxes” (107), emerging FMs could further obfuscate their inner workings, making the issue of explainability vital. Research addressing explainability will be invaluable for model advancement and deployment.

## 6. Conclusion

Dermatology presents both opportunities and challenges to integrate AI into its daily workings. Whilst in its infancy, with many regulatory standards that are specific to the field yet to be developed, current trajectories of innovation and advance showcase the potential of AI is likely to emerge a critical element of the dermatologists workflow, with the need for the clinician to have a global understanding of its workings. Steering this ship towards a future of a transparent, fair, safe, and responsible dermatology-AI will be an interdisciplinary effort that involves the leadership of the dermatology community.

## Author contributions

JO: Conceptualization, Project administration, Supervision, Writing – original draft, Writing – review & editing. HG: Conceptualization, Visualization, Writing – original draft, Writing – review & editing. RD: Resources, Supervision, Writing – review & editing. ZC: Supervision, Writing – original draft, Writing – review & editing. VM: Conceptualization, Resources, Supervision, Writing – review & editing.
